# Does need strength influence the impact of supervision styles on junior doctors’ affective states?

**DOI:** 10.1007/s10459-025-10491-y

**Published:** 2025-12-01

**Authors:** Wieke E. van der Goot, Robbert J. Duvivier

**Affiliations:** 1 Wenckebach Institute for Education and Training (WIOO), Lifelong Learning, Education and Assessment Research Network (LEARN), University of Groningen, University Medical Center Groningen, Groningen, the Netherlands; 2 Martini Hospital, Martini Academy, Groningen, the Netherlands; 3 Parnassia Psychiatric Institute, The Hague, The Netherlands

**Keywords:** Basic psychological needs, Need strength, Well-being, Self-determination theory, Postgraduate medical education, Supervision style

## Abstract

Junior doctors’ motivation and well-being benefit from a safe environment where they can practice with and learn from clinical experiences. Supervising consultants are key figures in this environment by providing safe patient care, but also by supporting junior doctors in their professional development and learning. However, many junior doctors suffer from distress, which suggests that their needs are not optimally met in postgraduate medical education. Self-Determination Theory (SDT) states that a social environment that supports basic psychological needs of learners results in better learning and promotes their well-being. However, there is a theoretical debate whether individual differences, such as need strength, may also influence how learners, such as junior doctors, perceive the environment. A better understanding of the combined role of the environment and individual preferences is relevant to tailor supervision styles to promote motivation and well-being of junior doctors. This secondary analysis of an experimental vignette study on (de)motivating supervision styles examined whether junior doctors’ individual differences in work-related need strength modified the appraisal and effects of consultants’ supervision styles. The analysis included 150 junior doctors (*M*_*age*_ = 29.81, 73.3% female) and showed that supervision style impacted junior doctors’ positive and negative affect through need satisfaction and need frustration, respectively. Junior doctors’ work-related need strength did not modify these effects. These findings underline the importance of considering both need satisfaction and frustration as underlying mechanisms for explaining well-being in Postgraduate Medical Education. Furthermore, the lack of moderation of work-related need strength endorses the principle of universality underlying SDT.

**Supplementary Information:**

The online version contains supplementary material available at https://doi.org/10.1007/s10459-025-10491-y.

In Postgraduate Medical Education (PGME), the dynamic interaction between junior doctors and their supervising consultants is essential for learning, professional development, and well-being. It can be challenging for supervising consultants to find a balance between ensuring safe patient care and creating an environment that motivates junior doctors to learn and supports their well-being. A better understanding of the influence of consultants’ supervision styles on junior doctors’ well-being is important because there is a relative high prevalence of distress and symptoms of burnout among junior doctors (Dyrbye et al., 2014; Prins et al., 2010; Ripp et al., 2017; van Vendeloo et al., 2018). Distress and burnout symptoms are likely related to a variety of negative professional and personal outcomes including reduced learning and performance, suboptimal quality of care, mental health issues, and drop out (Dyrbye & Shanafelt, 2016). From a Self-Determination Theory (SDT) perspective, these negative outcomes and ill-being can be a result of an environment, including supervision styles, that does not optimally support junior doctors’ needs (Ryan & Deci, 2000a). However, individual differences of junior doctors might also influence the effects of supervision styles on their motivation and well-being. In this study we examine the supervision relationship by considering both supervision styles and individual differences between junior doctors. We use SDT as a lens through which to examine this supervision relationship in PGME and provide guidance to promote motivating learning environments for junior doctors. 

## (De)Motivating supervision styles

Consultants play a central role in PGME by the way they provide supervision to junior doctors. Ideally, supervision is tailored to the competency level of the junior doctor and the specific characteristics of the clinical situation. It has been argued that clinical supervision can improve junior doctors’ learning and motivation if attention is paid to the style of supervision (Sawatsky et al., 2022). Research in other domains has shown that need-supportive styles are associated with need satisfaction and positive outcomes, including well-being (Aelterman et al., 2018; Baard et al., 2004; Deci et al., 1981; Delrue et al., 2019; Haerens et al., 2015, 2018; van der Kaap-Deeder et al., 2017; Vansteenkiste et al., 2019). Specifically, autonomy supportive styles focus on nurturing learners’ inner motivational resources, such as interests, preferences, and goals (Assor et al., 2002; Deci & Ryan, 2000; Reeve, 2009), while structuring styles provide clear expectations, guidelines, and tailored help (Jang et al., 2010; Skinner et al., 1998). In contrast, need-thwarting styles are associated with need frustration and negative outcomes, including ill-being (Aelterman et al., 2018; Deci et al., 1981; Delrue et al., 2019; Haerens et al., 2015, 2018; van der Kaap-Deeder et al., 2017; Vansteenkiste et al., 2019). Specifically, chaotic styles are characterised by lack of clear guidelines and expectations, permissiveness or even abandonment of learners (Aelterman et al., 2018), while controlling styles actively suppress autonomy and pressure learners to think, behave or feel in a specific way (Assor et al., 2002; Deci & Ryan, 2000; Jang et al., 2010).

In a previous study, we found that *high* need-supportive supervision styles enhanced junior doctors’ intrinsic motivation through need satisfaction, while *low* need-supportive styles (i.e., need-*thwarting* styles) diminished junior doctors’ intrinsic motivation through need frustration (van der Goot et al., 2025). In this secondary analysis, we examine both the positive and negative effects of (de)motivating supervision styles. We used affective state as our outcome variable, a situation specific emotional state and subjective well-being variable (Ryan & Deci, 2000b, 2001). Positive and negative affective states have been used before as indicators to psychological well-being and ill-being, respectively (Bartholomew et al., 2011). Anticipating similar effects on affective states, we hypothesised (*Hypothesis 1a*) that *high* (relative to *low*) need-supportive supervision styles positively predict positive affect through need satisfaction. Similarly, *Hypothesis 1b* states that *low* (relative to *high*) need-supportive supervision styles positively predict negative affect through need frustration. 

## Basic psychological needs and work-related need strength

SDT asserts that self-determination of behaviour is essential for professional development and well-being (Deci & Ryan, 2000, 2008; Vansteenkiste & Ryan, 2013). Self-determined behaviour is supported through satisfaction of the three basic psychological needs for autonomy, competence, and relatedness. The need for *autonomy* refers to the psychological need to experience volition and freedom of choice (DeCharms, 1968; Deci, 1975), *competence* refers to the psychological need to experience personal efficacy (Elliot et al., 2017; Harter, 1978; White, 1959), and *relatedness* refers to the psychological need to experience social connectedness with others (Baumeister & Leary, 1995; Harlow, 1958). Satisfaction and frustration of the basic psychological needs are underlying mechanisms to explain and predict positive and negative outcomes, including well-being and ill-being (Deci & Ryan, 2000; Ryan & Deci, 2000b, 2017; Vansteenkiste et al., 2010, 2020).

SDT’s three basic psychological needs are asserted to be innate and universal (Deci & Ryan, 2008). Regardless of individual differences, people’s well-being is assumed to be a function of the satisfaction (positive) and frustration (negative) of these needs (Deci & Ryan, 2008). Due to this principle of universality, individuals’ need strength (i.e., individual preferences for autonomy, competence, and relatedness) was not originally considered in SDT. However, it has been argued that no absolute uniformity is needed (Shweder & Sullivan, 1993). As a result, individual differences may also contribute to the perceived benefits and costs of need satisfaction and need frustration, respectively (Soenens et al., 2015; Van Assche et al., 2018; Vansteenkiste et al., 2020). In Motive Dispositions Theory (MDT; McClelland, 1965, 1985), individual differences in need strength are core constructs that are assumed to be learned and specific for a particular context (McClelland, 1965, 1985), including the workplace (Van Yperen et al., 2014; Wörtler et al., 2020). Translating this to SDT’s basic psychological needs of autonomy, competence, and relatedness it means that junior doctors may have different, learned preferences for each basic need. For instance, a higher need for autonomy (i.e., *higher* need strength) implies that junior doctors have a stronger preference that their autonomy is supported by supervising consultants. MDT’s matching hypothesis poses that people with a *higher* need strength would benefit more from satisfaction of the corresponding need than people with a *lower* need strength (Schüler et al., 2013).

**Fig. 1:**
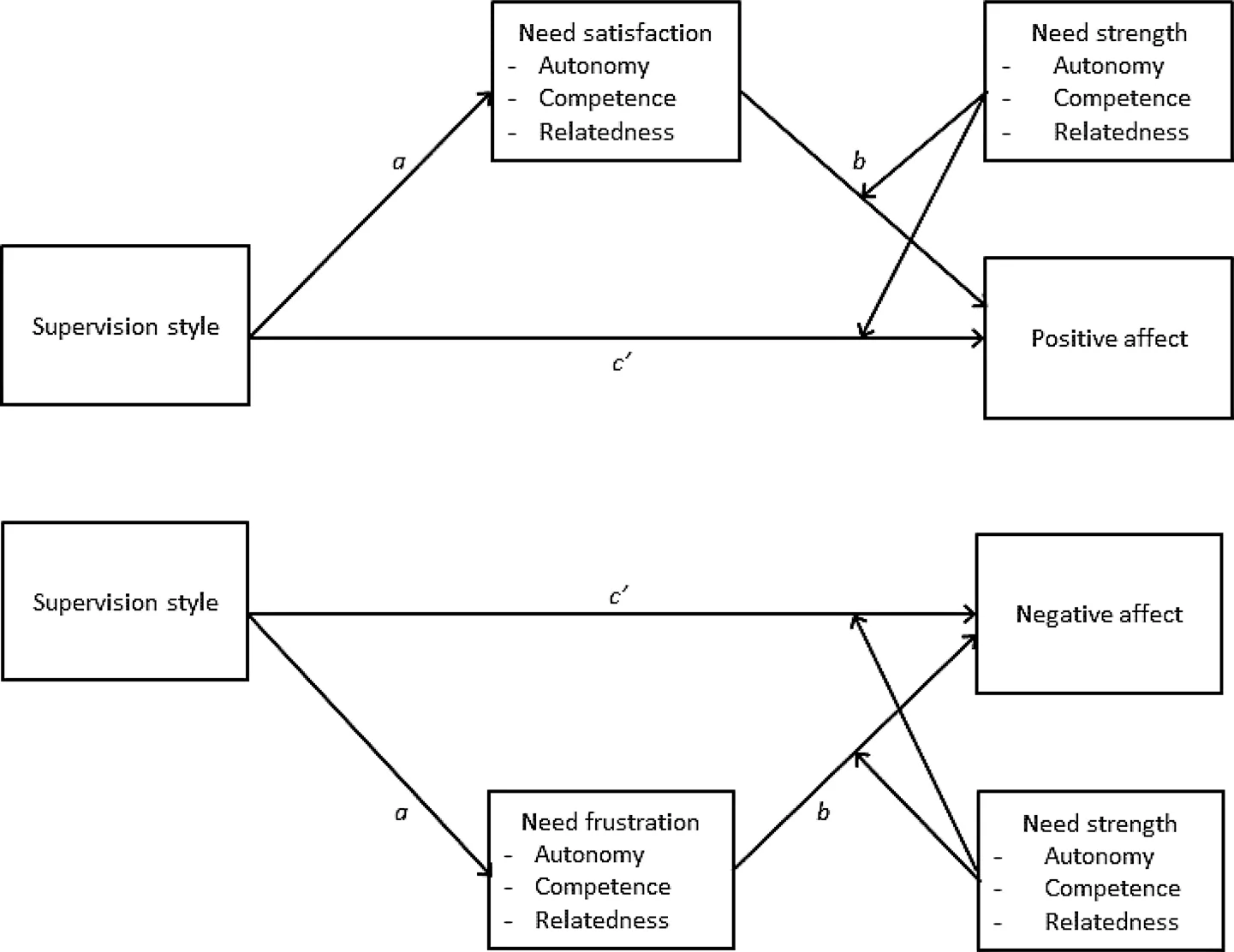
Conceptual model of second stage moderation of need strength on affective state. Note: This model shows second stage moderation of the effect of the mediator (i.e., need satisfaction and need frustration, respectively) on affective state. *a* = effect of supervision style on the mediator; *b* = effect of the mediator on affective state; *c’* = the direct effect of supervision style on affective state irrespective of the mediator and/or moderator

Individual differences in need strength may influence the effect of supervision styles on junior doctors’ affective states in two ways. First, in line with MDT’s matching hypothesis, need strength might strengthen the effects of need satisfaction and need frustration (Fig. 1). This requires a somewhat liberal view on SDT’s principle of universality (Soenens et al., 2015; Van Assche et al., 2018). Findings that support this hypothesis have been found for students’ autonomous motivation for homework (Katz et al., 2009), relatedness fulfilment and positive affect (Moller et al., 2010), and competence satisfaction and domain-specific flow and well-being (Schüler et al., 2013). However, other studies did not find (robust) moderating effects of need strength (e.g., Chen et al., 2015; Sheldon & Schüler, 2011). Limited and inconsistent effects of need strength were reported on need satisfaction, organisational citizenship behaviour, and work-engagement (Wörtler et al., 2020), and on autonomy satisfaction and frustration on well-being and ill-being, respectively (Van Assche et al., 2018). Because of these mixed findings it remains relevant to consider the possible moderating role of need strength in the context of PGME. Hence, we hypothesised that the positive direct effect between satisfaction of each need domain (i.e., autonomy, competence, and relatedness satisfaction) on positive affect would increase as junior doctors’ corresponding work-related need strength was higher (*Hypothesis 2a)*. Similarly, *Hypothesis 2b* is that the positive direct effect between frustration of each need domain on negative affect would increase as junior doctors’ corresponding work-related need strength was higher.

**Fig. 2:**
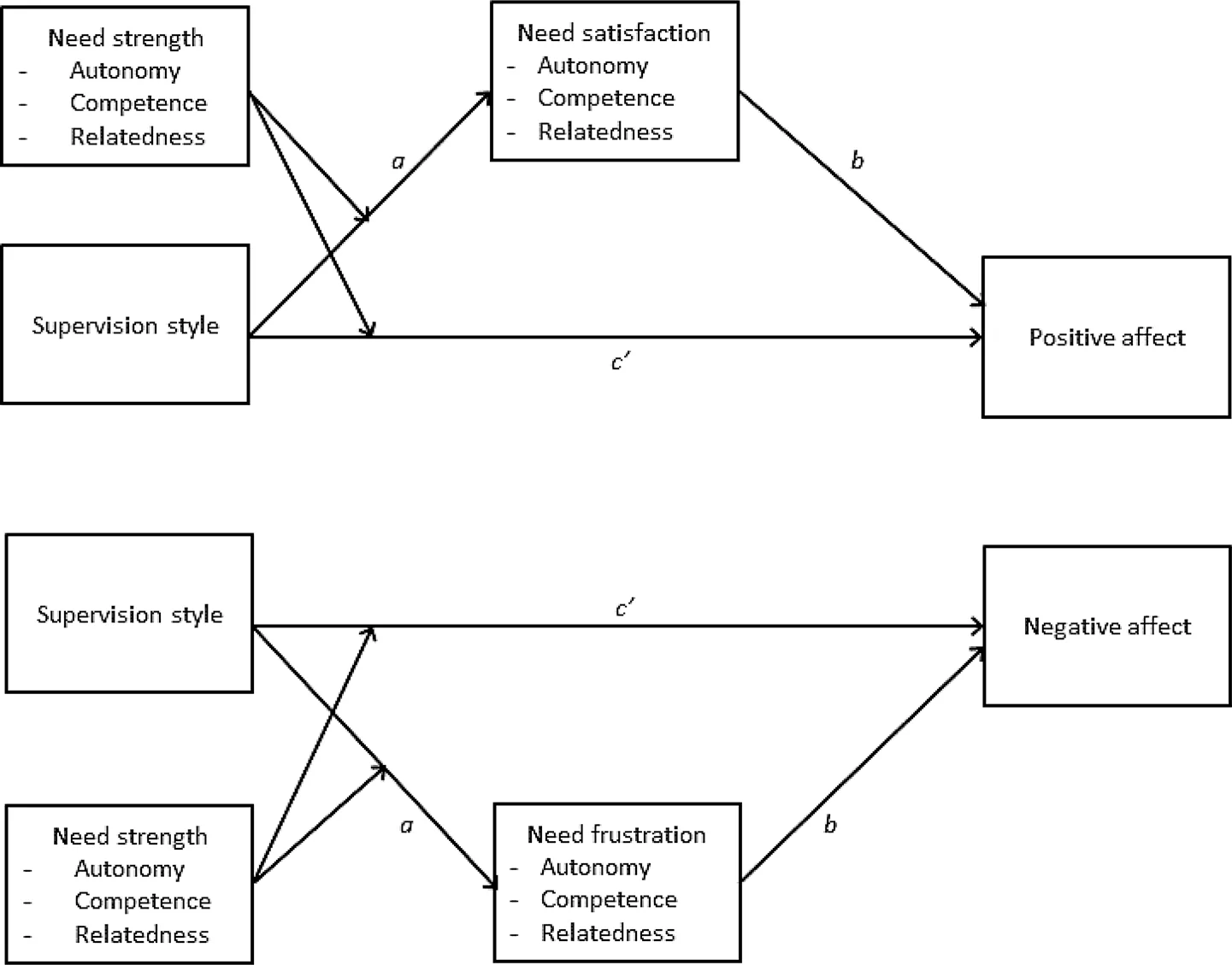
Conceptual model of first stage moderation of need strength on need domains and affective state. Note: This model shows first stage moderation of the effect of the supervision styles on the mediator (i.e., need satisfaction and need frustration, respectively). *a* = effect of supervision style on the mediator; *b* = effect of the mediator on affective state; *c’* = the direct effect of supervision style on affective state irrespective of the mediator and/or moderator

Second, individual differences in need strength might modify need-based experiences (Fig. 2). As consultants convey relevant clinical and professional values when they supervise, junior doctors may learn to interpret different supervision styles differently and develop work-related preferences for basic psychological needs. This reasoning is not at odds with SDT’s principle of universality. Within SDT, it has been acknowledged that the *functional significance* (i.e., the meaning) that people attach to (de)motivating behaviours (e.g., supervision styles) from the environment may result in different perceptions of these behaviours (Deci & Ryan, 1987; Soenens et al., 2015). For example, Baard et al. (2004) found that both autonomy support and individual causality orientation were positively associated with need satisfaction, and accordingly, with well-being and performance evaluations. Also, need strength was found to moderate perceived need support of teachers (Katz et al., 2009). Further, Sheldon and Schüler (2011) found that explicit motives for competence and relatedness predicted need satisfaction, and accordingly, affective states. Hence, we hypothesised that the positive direct effect of *high* (relative to *low*) need-supportive supervision on each psychological need, and accordingly, positive affect, would increase as junior doctors’ corresponding work-related need strength was higher (*Hypothesis 3a)*. Similarly, we hypothesised that the positive direct effect of *low* (relative to *high*) need-supportive supervision on each psychological need, and accordingly, negative affect, would increase as junior doctors’ corresponding work-related need strength was higher (*Hypothesis 3b)*.

## Method

### Procedure and participants

This research was a secondary analysis of an experimental vignette study among Dutch junior doctors on the effects of different (de)motivating supervision styles (see van der Goot et al., 2025). We specifically focussed on work-related need strength as a moderator and affective states as dependent variables. After providing informed consent, the participants indicated their work-related need for autonomy, competence, and relatedness. Next, they were randomly assigned to one of the four experimental conditions that differed in need support (*high* versus *low*) and directiveness (*high* versus *low*). The conditions were: *high* need support, *low* directiveness (autonomy supportive style), *high* need support, *high* directiveness (structuring style), *low* need support, *low* directiveness (chaotic style), and *low* need support, *high* directiveness (controlling style). In each condition, participants read four vignettes. The vignettes were short, written descriptions about clinical situations that junior doctors may encounter in practice. Only the supervision style (i.e., the manipulation) differed between the experimental conditions. After reading the vignettes, participants indicated their need satisfaction, need frustration, and affective states. Ethical approval for this study was obtained by the ethical board of the NVMO (Dutch Association for Medical Education; file #2020.7.1).

We recruited junior doctors who worked in University Medical Centres or teaching hospitals in the Netherlands. In this context, the term *junior doctor* refers to both residents in postgraduate specialty training and medical graduates not (yet) enrolled in training but fully licensed to practice. Both groups work under consultant supervision and regularly work in emergency departments, which formed the clinical setting for our vignettes.

We received 254 responses. Participants were excluded from analysis if their responses were incomplete (*n* = 100), the outlier analysis and close inspection of the answers revealed a suspect answer pattern (*n* = 3), or participants were identified as a multivariate outlier on the dependent variables (*n* = 1).

### Measures

#### Manipulation checks

As in van der Goot et al. (2025), we checked the manipulations through two subscales with three items each, namely ‘need support’ (α = 0.97), and ‘directiveness’ (α = 0.93). Items were rated on a 7-point Likert scale ranging from 1 (*not at* all) to 7 (*completely*). Supplementary Information 1 (SI1) shows the assessment of our manipulation checks.

#### Need strength

We assessed participants’ level of work-related need strength with a 12-item measure developed by Van Yperen et al. (2014). This scale measures need for autonomy (e.g., ‘I have the need to decide on my own how to go about getting my job done’), need for competence (e.g., ‘I have the need to feel skilled’), and need for relatedness (e.g., ‘I have the need to feel like I am part of a team or a group’) with four items each. Items were rated on a 7-point Likert scale ranging from 1 (*not at all*) to 7 (*to an extremely large extend*).

#### Basic psychological need frustration and satisfaction

Similar to van der Goot et al. (2025), participants’ autonomy, competence, and relatedness satisfaction and frustration were measured by the 24-item *Basic Psychological Need Satisfaction and Frustration* scale (four items per subscale) of Chen et al. (2015). Items were rated on a 5-point Likert scale ranging from 1 (*not true at all*) to 5 (*completely true*).

#### Affective state

We measured affective states with the 10-item version of the international PANAS (I-PANAS-SF; Thompson, 2007) that is based on the original 20-item PANAS (Watson et al., 1988). We adapted the stem of the scale to match the clinical context: ‘By the way in which my supervisor reacts in the four scenarios …’. This scale covers positive affect (e.g., ‘I feel inspired’) and negative affect (e.g., ‘I feel nervous’). Items were rated on a 5-point Likert scale ranging from 1 (*not true at all)* to 5 (*completely true)*.

## Statistical analyses

We used SPSS (IBM Corp, 2019; version 26) for descriptive analyses, manipulation checks (ANOVA), and the 2 × 2 MANOVA. To test our hypotheses, we used the PROCESS macro (version 4.3) developed for SPSS (Hayes, 2022). The PROCESS macro (Hayes, 2022) is a statistical tool based on Ordinary Least Squares (OLS) regression, which aims to estimate coefficients which best describe the linear relationship between independent (predictors) and dependent (outcome) variables. The tool allows testing of simple to highly complex mediation, moderation and moderated mediation models in which multiple variables are added to best describe the relationship between the independent (X), mediating (M), moderating (W), and dependent (Y) variables. See SI2 for the graphical and statistical models that were tested in this study. In all analyses we used a heteroscedasticity-consistent estimator (HC4) to reduce bias due to heteroscedasticity in the error variances (Cribari-Neto, 2004). To include our categorical independent variable (supervision styles) in the regression models, we used contrast coding to dummy code the four supervision styles (West et al., 1996). The four supervision styles were translated into three dummy codes (D_1_ – D_3_) with separate regression coefficients. The first and most important dummy (D_1_) contrasted *high* and *low* need-supportive supervision styles. This dummy was used to test the hypothesised effects of *high* (relative to *low*) need-supportive supervision styles in *Hypotheses 1a, 2a,* and *3a*. We reversed the contrast codes of D_1_ in *Hypotheses 1b, 2b,* and *3b* to align it with our focus on *low* (relative to *high*) need-supportive supervision styles. The second dummy (D_2_), contrasted both *high* need-supportive supervision styles, that differed in directiveness. Finally, the third dummy (D_3_), contrasted both *low* need-supportive supervision styles, that differed in directiveness. All models computed direct (*b* and *c’* coefficients) and indirect effects (*ab* coefficients) of supervision styles on positive or negative affect. The *b-*path is the estimated coefficient that represents the effect of the mediator on positive or negative affect, while the *c’-*path is the effect of supervision style on affective state irrespective of the mediator and/or moderator. The *ab-*path estimates the indirect effect between supervision style (D_1_ – D_3_) on positive or negative affect.

To test *Hypotheses 1a* and *1b*, we selected Hayes (2022) PROCESS Model 4 with two parallel mediators to test for unmoderated mediation effects of supervision styles on affective states through need satisfaction and need frustration. This simple parallel mediation model tested if supervision styles predicted positive and negative affect through need satisfaction and need frustration, respectively. We used composite scores of need satisfaction (i.e., summarised mean values of all autonomy, competence, and relatedness satisfaction items) and need frustration (i.e., summarised mean values of all autonomy, competence, and relatedness frustration items) to combine the predictive values of the individual needs while simultaneously reducing the effects of multiple testing and multicollinearity (as indicated by the relatively high correlations between the basic psychological needs in Table 1). The use of composite scores of need satisfaction and need frustration was used in previous studies (e.g., Bartholomew et al., 2011; Chen et al., 2015; van der Kaap-Deeder et al., 2017).

**Table 1:** Cronbach’s alpha’s, means, standard deviations, and correlations of the continuous variables

Variable	α	M	SD	1	2	3	4	5	6	7	8	9	10	11
1. Autonomy satisfaction	0.87	3.19	0.92	–										
2. Competence satisfaction	0.96	3.53	1.14	0.71**	–									
3. Relatedness satisfaction	0.93	3.10	0.91	0.60**	0.69**	–								
4. Autonomy frustration	0.87	2.17	0.98	−0.50**	−0.71**	−0.53**	–							
5. Competence frustration	0.94	2.15	1.15	−0.50**	−0.72**	−0.54**	0.72**	–						
6. Relatedness frustration	0.94	1.91	1.06	−0.51**	−0.71**	−0.62**	0.69**	0.74**	–					
7. Positive affect	0.75	3.26	0.76	0.56**	0.66**	0.53**	−0.48**	−0.59**	−0.59**	–				
8. Negative affect	0.90	2.31	1.02	−0.52**	−0.78**	−0.59**	0.76**	0.83**	0.73**	−0.59**	–			
9. Need for autonomy	0.87	4.72	0.81	−0.08	−0.09	−0.05	0.12	0.03	0.15	−0.08	0.02	–		
10. Need for competence	0.85	5.64	0.60	0.00	0.02	−0.07	0.02	0.04	0.02	0.03	−0.04	0.37**	–	
11. Need for relatedness	0.85	4.57	0.97	0.00	−0.05	0.01	0.07	−0.00	0.03	0.07	0.07	0.33**	0.25*	–

*Notes. α = Cronbach’s α, M = mean, SD = standard deviation.* *p < 0.05. **p < 0.001

For *Hypothesis 2a* and *2b* we selected Hayes (2022) PROCESS Model 15. This model represents second stage moderation of the effect of the mediator (i.e., need domain) on positive or negative affect, and moderation of the direct effect of supervision style (D_1_ – D_3_) on affect (see Fig. 1). For *Hypotheses 3a* and *3b* we selected Hayes (2022) PROCESS Model 8. This model represents first stage moderation of the effect of supervision style (D_1_ – D_3_) on the mediator (i.e., need domain), and moderation of the direct effect of supervision style (D_1_ – D_3_) on affect (see Fig. 2). The models for each need domain (i.e., autonomy, competence, and relatedness) were calculated separately. The rationale behind this is the theory driven assumption that, *if there is an effect,* need for autonomy should influence autonomy satisfaction and frustration, but not the other need domains (competence and relatedness). The same theoretical assumptions underly need for competence with competence satisfaction and frustration, and need for relatedness for relatedness satisfaction and frustration. Both the psychological need variables (Model 15) and the moderator variables were mean centred (Model 8 and Model 15). The index of moderated mediation (*ab-*path) of each dummy variable (D_1_ – D_3_) was interpreted to identify if moderated mediation was present (Hayes, 2015, 2022). If at least one of these effects was significantly different from zero, we inspected how the indirect relations were different for different levels of the moderator variable.

## Results

Our sample comprised 150 participants (73.3% female). Participants’ ages ranged from 25 to 42 (*M* = 29.81, *SD* = 3.06). Participants worked in 22 different specialties; 53 (35.3%) were junior doctors not-in-training and 97 (64.7%) were trainees. Trainees were in postgraduate year (PGY) 1–2 (*n* = 46, 46.5%), PGY 3–4 (*n* = 34, 34.4%) or PGY 5–6 (*n* = 19, 19.2%).

### Correlations and descriptive statistics

Table 1 shows the means, standard deviations and correlations among the continuous variables. Affect was significantly correlated with need satisfaction and need frustration, but not with work-related need strength.

### Hypothesis testing

In *Hypothesis 1a* we predicted that a positive indirect relationship exists between *high* (relative to *low*) need-supportive supervision styles and positive affect through need satisfaction. Using Hayes (2022) PROCESS Model 4, the results revealed that positive affect was significantly predicted by need satisfaction (*b*_*1*_ = 0.31, *SE* = 0.11, 95% CI 0.08 to 0.53), but not by need frustration (*b*_*2*_ = −0.11, *SE* = 0.09, 95% CI −0.29 to 0.07), *R*^*2*^ = 0.51, *F*(5, 144) = 34.79, *p* < 0.001. The indirect effect of *high* (relative to *low*) need-supportive supervision (D_1_) on positive affect was significantly explained through need satisfaction (*a*_*1*_*b*_*1*_ = 0.40, *SE* = 0.15, 95% CI 0.12 to 0.69), but not through need frustration (*a*_*4*_*b*_*2*_ = 0.16, *SE* = 0.13, 95% CI −0.10 to 0.39). We found no significant indirect effects of *high* need-supportive supervision styles that differed in directiveness (D_2_) and *low* need-supportive supervision styles that differed in directiveness (D_3_) on positive affect. Thus, we concluded that only need satisfaction mediated the effect of *high* (relative to *low*) need-supportive supervision on positive affect, which provided empirical support for *Hypothesis 1a* (for all indirect effects, see SI3).

In *Hypothesis 1b,* we posed that a positive indirect relation exists between *low* (relative to *high*) need-supportive supervision styles and negative affect through need frustration. Similar PROCESS analysis (Hayes, 2022) revealed that negative affect was significantly predicted by need frustration (*b*_*2*_ = 0.63, *SE* = 0.08, 95% CI 0.47 to 0.78), but not by need satisfaction (*b*_*1*_ = −0.08, SE = 0.08, 95% CI −0.24 to 0.07), *R*^*2*^ = 0.79, *F*(5, 144) = 121.16, *p* < 0.001. The relative indirect effect of *low* (relative to *high*) need-supportive supervision (D_1 r_) on negative affect was significantly explained through need frustration (*a*_*4*_*b*_*2*_ = 0.88, *SE* = 0.12, 95% CI 0.65 to 1.12), but not through need satisfaction (*a*_*1*_*b*_*1*_ = 0.10, *SE* = 0.10, 95% CI −0.09 to 0.31). We found no significant indirect effects of *high* need-supportive supervision styles that differed in directiveness (D_2_) and *low* need-supportive supervision styles that differed in directiveness (D_3_) on negative affect. Thus, we concluded that only need frustration mediated the effect of *low* (relative to *high*) need-supportive supervision on negative affect (see SI3), which provided empirical support for *Hypothesis 1b*.

An additional Multivariate Analysis of Variance (MANOVA) revealed the anticipated multivariate main effect of need support, *F*(8, 139) = 39.71, *p* < 0.001, $\eta _p^2$ = 0.70 (see SI4). Table 2 shows that participants in the *high* need support conditions scored higher on all need satisfaction measures and positive affect whereas participants in the *low* need support conditions scored higher on all need frustration measures and negative affect.

**Table 2:** Means and standard deviations between high and low need-supportive supervision styles

	*High* need support	*Low* need support
Variables	*M (SD)*	*M (SD)*
1. Autonomy satisfaction	3.67 (0.79)	2.68 (0.77)
2. Competence satisfaction	4.35 (0.66)	2.66 (0.87)
3. Relatedness satisfaction	3.66 (0.73)	2.52 (0.70)
4. Autonomy frustration	1.52 (0.65)	2.86 (0.78)
5. Competence frustration	1.43 (0.66)	2.92 (1.05)
6. Relatedness frustration	1.23 (0.54)	2.62 (1.01)
7. Positive affect	3.71 (0.56)	2.77 (0.63)
8. Negative affect	1.54 (0.60)	3.13 (0.69)

*Note.* Within each row, means differ by *p* < 0.001

In *Hypothesis 2a,* we predicted that the positive direct effect between satisfaction of each need domain on positive affect increased if junior doctors’ corresponding work-related need strength increased. Using Hayes (2022) PROCESS Model 15, the results shown in the upper half of Table 3 revealed that all relative indirect effects (*ab-*paths, the indices of moderated mediation) were not significant. Thus, we found no empirical support for *Hypothesis 2a,* meaning that the effects of each need domain on positive affect were not moderated by each corresponding need strength variable.

**Table 3:** Indirect and direct effects of supervision styles on affective states through need satisfaction and need frustration, with work-related need strength as moderator (Model 15)

Variable	Antecedent	Relative indirect effect				Direct effect						*R*^*2*^	*R*^*2*^_*change*_
			Coeff.	SE	99% CI			Coeff.	SE	99% CI			
					*LL*	*UL*				*LL*	*UL*		
Positive affect													
AutSat												0.51***	
	D_1_ X NfAut	*a*_*1*_*b*_*2*_	−0.12	0.11	−0.40	0.17	*c*_*1*_’	−0.03	0.20	−0.55	0.44		0.01^a^
	D_2_ X NfAut	*a*_*2*_*b*_*2*_	−0.05	0.05	−0.22	0.06	*c*_*2*_’	0.02	0.16	−0.41	0.42		
	D_3_ X NfAut	*a*_*3*_*b*_*2*_	−0.03	0.04	−0.17	0.06	*c*_*3*_’	−0.30	0.19	−0.84	0.19		
	M (AutSat)						*b*_*1*_	0.25***	0.07	0.07	0.42		
	AutSat X NfAut						*b*_*2*_	−0.11	0.11	−0.38	0.16		0.01^b^
ComSat												0.53***	
	D_1_ X NfCom	*a*_*1*_*b*_*2*_	0.28	0.19	−0.26	0.80	*c*_*1*_’	−0.25	0.27	−0.89	0.55		0.02^a^
	D_2_ X NfCom	*a*_*2*_*b*_*2*_	0.03	0.03	−0.04	0.16	*c*_*2*_’	−0.03	0.20	−0.58	0.49		
	D_3_ X NfCom	*a*_*3*_*b*_*2*_	0.05	0.05	−0.08	0.22	*c*_*3*_’	−0.43	0.20	−0.88	0.19		
	M (ComSat)						*b*_*1*_	0.26**	0.08	0.05	0.44		
	ComSat X NfCom						*b*_*2*_	0.16	0.11	−0.15	0.45		0.01^b^
RelSat												0.49***	
	D_1_ X NfRel	*a*_*1*_*b*_*2*_	0.03	0.08	−0.17	0.24	*c*_*1*_’	−0.15	0.15	−0.51	0.25		0.02^a^
	D_2_ X NfRel	*a*_*2*_*b*_*2*_	0.01	0.02	−0.05	0.07	*c*_*2*_’	0.11	0.13	−0.22	0.47		
	D_3_ X NfRel	*a*_*3*_*b*_*2*_	−0.00	0.01	−0.05	0.04	*c*_*3*_’	−0.28	0.13	−0.59	0.13		
	M (RelSat)						*b*_*1*_	0.19**	0.07	0.03	0.36		
	RelSat X NfRel						*b*_*2*_	0.03	0.07	−0.15	0.20		0.00^b^
Negative affect													
AutFru												0.73***	
	D_1 reversed_ X NfAut	*a*_*1*_*b*_*2*_	−0.18	0.12	−0.54	0.10	*c*_*1*_’	0.26	0.17	−0.17	0.73		0.01^a^
	D_2_ X NfAut	*a*_*2*_*b*_*2*_	−0.02	0.03	−0.13	0.04	*c*_*2*_’	−0.15	0.15	−0.51	0.28		
	D_3_ X NfAut	*a*_*3*_*b*_*2*_	−0.03	0.03	−0.14	0.05	*c*_*3*_’	0.04	0.18	−0.47	0.53		
	M (AutFru)						*b*_*1*_	0.49***	0.09	0.26	0.70		
	AutFru X NfAut						*b*_*2*_	−0.13	0.09	−0.38	0.08		0.01^b^
ComFru												0.80***	
	D_1 reversed_ X NfCom	*a*_*1*_*b*_*2*_	−0.03	0.15	−0.42	0.40	*c*_*1*_’	0.04	0.24	−0.64	0.62		0.00^a^
	D_2_ X NfCom	*a*_*2*_*b*_*2*_	−0.00	0.02	−0.07	0.08	*c*_*2*_’	−0.12	0.16	−0.51	0.34		
	D_3_ X NfCom	*a*_*3*_*b*_*2*_	0.01	0.07	−0.19	0.22	*c*_*3*_’	−0.06	0.22	−0.66	0.49		
	M (ComFru)						*b*_*1*_	0.51***	0.06	0.35	0.64		
	ComFru X NfCom						*b*_*2*_	−0.02	0.10	−0.28	0.25		0.00^b^
RelFru												0.71***	
	D_1 reversed_ X NfRel	*a*_*1*_*b*_*2*_	0.04	0.09	−0.19	0.33	*c*_*1*_’	0.08	0.16	−0.34	0.51		0.01^a^
	D_2_ X NfRel	*a*_*2*_*b*_*2*_	0.01	0.02	−0.04	0.07	*c*_*2*_’	−0.16	0.12	−0.55	0.11		
	D_3_ X NfRel	*a*_*3*_*b*_*2*_	−0.01	0.03	−0.11	0.06	*c*_*3*_’	0.23	0.15	−0.13	0.64		
	M (RelFru)						*b*_*1*_	0.36***	0.07	0.17	0.55		
	RelFru X NfRel						*b*_*2*_	0.03	0.07	−0.14	0.23		0.00^b^

*Note.* All regression coefficients are unstandardised. The standard error (SE) and 99% confidence intervals (CI) are bootstrapped with 10,000 samples. *LL* = lower limit; *UL* = upper limit. (In)direct effects are considered significant when the parameters of the Lower Limit (*LL*) and Upper Limit (*UL*) do not include zero. D_1_ = difference between *high* and *low* need-supportive supervision styles; D_2_ = difference between both *high* need-supportive supervision styles; D_3_ = difference between both *low* need-supportive styles; D_1 reversed_ = difference between *low* and *high* need-supportive supervision styles. AutSat = autonomy satisfaction; ComSat = competences satisfaction; RelSat = relatedness satisfaction; AutFru = autonomy frustration; ComFru = competence frustration; RelFru = relatedness frustration; NfAut = Need for Autonomy; NfCom = Need for Competence; NfRel = Need for Relatedness. The relative indirect effects, represented as *ab*-paths, represent the indices for moderated mediation. The relative indirect effects, represented as *ab*-paths, represent the indices for moderated mediation.*R*^*2*^_*change*_ = the added explained variance by adding the moderator variable to the model. *R*^*2*^_*change*_^a^ refers to the impact of the moderator on c-paths (X on Y), while *R*^*2*^_*change*_^b^ refers to the b-path (M on Y). Due to multiple testing, we used a Bonferroni correction and considered *p* < 0.008 as significant. A heteroscedasticity consistent estimator was used in the analyses and to calculate the *p*-values. ** *p* < 0.008, ***p < 0.001

In *Hypothesis 2b*, we predicted that the positive direct effect between frustration of each need domain on negative affect increased if junior doctors’ corresponding work-related need strength increased. The same PROCESS analyses (Hayes, 2022), shown in the lower half of Table 3, revealed that all relative indirect effects (*ab-*paths, the indices of moderated mediation) were not significant. Thus, we found no empirical support for *Hypothesis 2b*, meaning that the effects of each need domain on negative affect were not moderated by each corresponding need strength variable.

In *Hypothesis 3a*, we predicted that the positive direct effect of *high* (relative to *low*) need-supportive supervision on each need domain and positive affect increased if junior doctors’ corresponding work-related need strength increased. Using Hayes (2022) PROCESS Model 8, the results shown in the upper half of Table 4 revealed that all relative indirect effects (*ab-*paths, indices of moderated mediation) were not significant. Hence, we found no empirical support for *Hypothesis 3a*. That is, individual differences in need strength of junior doctors did not moderate the effect of supervision style on positive affect through need satisfaction.

**Table 4:** Indirect and direct effects of supervision styles on affective states through need satisfaction and need frustration, with work-related need strength as moderator (Model 8)

Variable	Antecedent	Relative indirect effect					Direct effect					*R*^*2*^	*R*^*2*^_*change*_
			Coeff.	SE	99% CI			Coeff.	SE	99% CI			
					*LL*	*UL*				*LL*	*UL*		
Positive affect													
AutSat												0.50***	0.02
	*D*_*1*_ *X NfAut*	*a*_*5*_*b*	0.03	0.04	−0.06	0.18	*c*_*5*_’	−0.15	0.13	−0.52	0.15		
	*D*_*2*_*X NfAut*	*a*_*6*_*b*	0.04	0.05	−0.11	0.19	*c*_*6*_’	−0.04	0.17	−0.48	0.41		
	*D*_*3*_ *X NfAut*	*a*_*7*_*b*	−0.01	0.06	−0.15	0.21	*c*_*7*_’	−0.32	0.18	−0.84	0.14		
	*M* (AutSat)						*b*	0.24**	0.07	0.05	0.42		
ComSat												0.52***	0.02
	*D*_*1*_ *X NfCom*	*a*_*5*_*b*	−0.02	0.05	−0.19	0.12	*c*_*5*_’	0.07	0.14	−0.30	0.44		
	*D*_*2*_ *X NfCom*	*a*_*6*_*b*	−0.07	0.06	−0.26	0.09	*c*_*6*_’	−0.02	0.20	−0.57	0.51		
	*D*_*3*_ *X NfCom*	*a*_*7*_*b*	−0.04	0.09	−0.30	0.20	*c*_*7*_’	−0.44	0.20	−0.88	0.18		
	*M* (ComSat)						*b*	0.26**	0.08	0.05	0.44		
RelSat												0.48***	0.02
	*D*_*1*_ *X NfRel*	*a*_*5*_*b*	0.02	0.03	−0.05	0.11	*c*_*5*_’	−0.10	0.09	−0.33	0.14		
	*D*_*2*_ *X NfRel*	*a*_*6*_*b*	−0.04	0.04	−0.15	0.08	*c*_*6*_’	0.13	0.13	−0.21	0.47		
	*D*_*3*_ *X NfRel*	*a*_*7*_*b*	−0.03	0.04	−0.15	0.06	*c*_*7*_’	−0.27	0.13	−0.57	0.11		
	*M* (RelSat)						*b*	0.19**	0.06	0.02	0.36		
Negative affect													
AutFru												0.73***	0.002
	*D*_*1*_ *X NfAut*	*a*_*5*_*b*	0.09	0.08	−0.12	0.32	*c*_*5*_’	0.07	0.12	−0.25	0.39		
	*D*_*2*_ *X NfAut*	*a*_*6*_*b*	0.00	0.08	−0.24	0.22	*c*_*6*_’	−0.16	0.15	−0.53	0.27		
	*D*_*3*_ *X NfAut*	*a*_*7*_*b*	0.03	0.13	−0.33	0.45	*c*_*7*_’	0.03	0.18	−0.48	0.49		
	*M* (AutFru)						*b*	0.47***	0.08	0.25	0.68		
ComFru												0.80***	0.001
	*D*_*1*_ *X NfCom*	*a*_*5*_*b*	0.05	0.12	−0.25	0.39	*c*_*5*_’	0.00	0.13	−0.36	0.32		
	*D*_*2*_ *X NfCom*	*a*_*6*_*b*	0.14	0.15	−0.23	0.59	*c*_*6*_’	−0.12	0.15	−0.51	0.34		
	*D*_*3*_ *X NfCom*	*a*_*7*_*b*	−0.17	0.19	−0.72	0.31	*c*_*7*_’	−0.05	0.21	−0.61	0.49		
	*M* (ComFru)						*b*	0.51***	0.06	0.35	0.64		
RelFru												0.71***	0.01
	*D*_*1*_ *X NfRel*	*a*_*5*_*b*	−0.02	0.06	−0.20	0.12	*c*_*5*_’	0.13	0.09	−0.08	0.39		
	*D*_*2*_ *X NfRel*	*a*_*6*_*b*	0.00	0.04	−0.14	0.12	*c*_*6*_’	−0.15	0.12	−0.52	0.12		
	*D*_*3*_ *X NfRel*	*a*_*7*_*b*	−0.00	0.11	−0.29	0.31	*c*_*7*_’	0.20	0.14	−0.17	0.59		
	*M* (RelFru)						*b*	0.36***	0.07	0.18	0.56		

*Note.* All regression coefficients are unstandardised. All need strength variables were mean centred prior to analysis. A heteroscedasticity consistent estimator was used in the analyses and to calculate the p-values. The standard error (SE) and 99% confidence intervals (CI) are bootstrapped with 10,000 samples. *LL* = lower limit; *UL* = upper limit. (In)direct effects are considered significant when the parameters of the Lower Limit (*LL*) and Upper Limit (*UL*) do not include zero. D_1_ = difference between *high* and *low* need-supportive supervision styles; D_2_ = difference between both *high* need-supportive supervision styles; D_3_ = difference between both *low* need-supportive styles; D_1 reversed_ = difference between *low* and *high* need-supportive supervision styles. AutSat = autonomy satisfaction; ComSat = competences satisfaction; RelSat = relatedness satisfaction; AutFru = autonomy frustration; ComFru = competence frustration; RelFru = relatedness frustration; NfAut = Need for Autonomy; NfCom = Need for Competence; NfRel = Need for Relatedness. The relative indirect effects, represented as *ab*-paths, represent the indices for moderated mediation.*R*^*2*^_*change*_ = the added explained variance by adding the moderator variable to the model. Due to multiple testing, we used a Bonferroni correction and considered *p* < 0.008 as significant. ** *p* < 0.008,***p < 0.001

Finally, in *Hypothesis 3b*, we predicted that the positive direct effect of *low* (relative to *high*) need-supportive supervision on each corresponding psychological need and negative affect increased if junior doctors’ corresponding work-related need strength increased. The same PROCESS analyses (Hayes, 2022), shown in the lower half of Table 4, revealed all relative indirect effects (*ab-*paths, indices of moderated mediation) were not significant. Hence, we found no empirical support for *Hypothesis 3b*. That is, individual differences in need strength of junior doctors did not moderate the effect of supervision style on negative affect through need frustration.

## Discussion

In this study, we tested whether individual differences in work-related need strength modified the perception or appraisal of different (de)motivating supervision styles. Contrary to our hypothesis, we found no evidence that work-related need strength modified the effect of need-based experiences on affective states. Nor that work-related need strength modified the effect of supervision styles on need-based experiences. Thus, we found empirical support for SDT’s principle of universality (Deci & Ryan, 2000, 2008) rather than for MDT’s matching hypothesis (cf., Schüler et al., 2013; Sheldon & Schüler, 2011). Our findings illustrate that junior doctors’ affective states benefit from need satisfaction and suffer from need frustration due to different supervision styles, regardless their work-related need strength.

### Theoretical considerations

Some theoretical considerations need to be addressed. First, the role of need strength is debated and its operationalisation differs (Sheldon & Gunz, 2009; Sheldon & Schüler, 2011). In this research we operationalised need strength as *explicit*, context-specific individual difference variables. We took a neutral stance in the developmental origin of need strength, but reasoned that people may differ in their learned preference for specific needs (Katz et al., 2009). Our measurement of need strength was similar to previous studies (e.g., Van Yperen et al., 2014; Wörtler et al., 2020) and relied on SDT’s basic psychological needs (i.e., autonomy, competence, and relatedness) instead of MDT’s motives (i.e., power, achievement, and affiliation). It is posited that *explicit* motives are learned later in life and are cognition-based. Therefore, *explicit* measures of need strength often rely on self-report measures that assess the self-attributed motives of a person (Brunstein et al., 2018; McClelland et al., 1989). However, previous studies have also used *implicit* measures of need strength. It is posited that *implicit* motives develop early in life as a result of affective experiences Therefore, *implicit* measures often rely on non-conscious motives that are assessed indirectly, for example, through picture-story exercises (Brunstein et al., 2018; McClelland et al., 1989). These differences in operationalisation may result in different outcomes. In this study, we deemed an *explicit* operationalisation to be better aligned with SDT’s basic psychological needs and the most straightforward way to test the impact of individual differences of importance of autonomy, competence, and relatedness in junior doctors. Moreover, the *explicit* measurement is better aligned with the impact of only a limited number of situations (i.e., vignettes) that were assessed in this study. Despite the differences in operationalising need-strength, our findings were congruent with previous research indicating that both *explicit* and *implicit* need strength did not contribute substantially above and beyond the effects of need-based experiences (Chen et al., 2015; Van Assche et al., 2018; Wörtler et al., 2020). This is consistent with SDT’s reasoning that need strength, among other variables, can be a function of individuals’ developmental history, while need-based experiences influence human functioning (Ryan & Vansteenkiste, 2023). It is of practical relevance that individual preferences for autonomy, competence, and relatedness do not seem to act as catalysts for the positive effects of need satisfaction or as buffers for the negative effects of need frustration. Nevertheless, this assumption should be tested in clinical contexts to assess its generalisability to actual practice.

Second, SDT’s dual-process model of human functioning posits that the effects of need satisfaction and need frustration differ (Bartholomew et al., 2011; Costa et al., 2015; Haerens et al., 2015), and both concepts may uniquely predict motivational outcomes including well-being, ill-being, and (maladaptive) functioning (Deci & Ryan, 2000; Vansteenkiste & Ryan, 2013). In this research, the *high* need-supportive supervision styles explained approximately half of the variance in positive affect, while *low* need-supportive supervision styles explained nearly three quarters of the variance in negative affect. Thus, we found support for SDT’s dual-process model.

Third, our use of composite scores of need satisfaction and need frustration, instead of the individual need-based constructs, may be considered a conceptual limitation. Within SDT, the three basic psychological needs are theoretically distinct and expected to exert unique effects on motivational outcomes. Although they may co-vary in interpersonal interactions (Vansteenkiste et al., 2010) and are interrelated within specific situations or domains (Ryan & Deci, 2017), it remains important to examine them separately. Future research should further clarify the value of both composite and distinct need constructs in different clinical contexts.

### Practical implications

Our findings underline the importance of consultants knowing how to adopt need-supportive styles and avoid need-frustrating styles. Previous studies indicated that need-supportive styles can be trained (e.g., Hardré & Reeve, 2009; Neufeld, 2021; Reeve, 1998; Reeve & Cheon, 2021; Vansteenkiste et al., 2019, 2020), but supervisors’ beliefs and motivation are important to adopt need-supportive styles in practice (Reeve et al., 2014; Vermote et al., 2020). Therefore, we recommend sharing theoretical knowledge about SDT’s basic psychological needs with supervising consultants for discussion and reflection on their supervisory interactions with junior doctors. The vignettes can be used for training to illustrate how supporting (*versus* thwarting) these needs can promote autonomous (*versus* controlled) motivation and well-being (*versus* ill-being) in clinical practice. Furthermore, the vignettes show that relatively small changes in how supervising consultants frame their supervision already can have a positive impact. The use of inviting, supportive language (e.g., ‘can’, ‘may’, ‘could’) instead of controlling language (e.g., ‘must’, ‘need’, ‘should’) can already help to reduce pressure on junior doctors to behave in specific ways (Kusurkar et al., 2011; Neufeld, 2021). We would also suggest that short moments of briefing and debriefing before and after shifts may help to build mutual understanding and trust between supervisors and junior doctors (e.g., knowing if there are competing tasks for both parties during the shift and a personal check-in). These (de)briefings can also explore and align expectations and focus on how supervising consultants can best support the junior doctor in instances of doubt (e.g., in what situations do you want me to guide you through the process and when would it be more helpful if I encourage you to find a solution yourself?). Practical tips for implementing need-supportive styles in clinical practice are, for instance, to provide junior doctors with choices and responsibilities that are aligned with their interests and appropriate to the specific circumstances, to provide rationales for clinical decision making (autonomy), to provide structured guidance (scaffolding) and match clinical reasoning questions with junior doctors’ current skills and knowledge (competence), and to build a warm and trusting relationship by being empathic to junior doctors’ (learning) challenges and sharing personal experiences (relatedness). Thus, it is important to take the perspective of junior doctors as learners and aim to understand and acknowledge their viewpoints, values, and emotions.

### Strengths and limitations

A strength of this research is that we used an experimental design with vignettes that allowed us to draw conclusions based on causal relationships. Our models showed that supervision styles predicted positive affect especially through need satisfaction, and negative affect through need frustration. Furthermore, we tested the moderating role of need strength on need satisfaction and need frustration, with separate models for each need domain. This allowed us to compare if need strength modified the bright pathway towards positive affect through need satisfaction and the dark pathway towards negative affect through need frustration. Two limitations of our design need to be addressed. First, our vignettes could not assess long-term impact of supervision styles. The long-term impact of experiencing (different) supervision styles needs to be addressed in future research. Second, we found an unexpected limited impact of the directiveness dimension compared to the need-support dimension. In line with our expectations, participants were able to correctly identify differences in need-support and directiveness of the different supervision styles in the manipulation checks (SI1). However, the interaction effect of the MANOVA (SI4) that illustrated the effects of supervision styles showed an overwhelming impact of need support on our outcome variables (Table 2), but this was not found for directiveness and the interaction between need support and directiveness. Perhaps the role of directiveness is smaller or more nuanced. This could be examined in future research. From a practical stance, the impact of need thwarting styles that differ in directiveness may have different negative emotional outcomes for junior doctors. For example, junior doctors may experience fear and uncertainty if they feel they are left on their own (chaotic style), or frustration and anger if they feel pressured (controlling style). Moreover, a *high* directive and need-supportive approach (structuring style) may be well aligned with expectations of inexperienced junior doctors, but it seems not so well aligned with experienced junior doctors.

Another limitation of our study is that all constructs were self-reported (Podsakoff et al., 2003) meaning they may not cover all relevant aspects of clinical practice. Future studies may examine these constructs from multiple sources, for example combining self-report measures with observations, interviews or educational climate measures. Furthermore, understanding the affective and emotional outcomes of supervisory interactions may benefit from (a) qualitative methods, such as rich pictures, that capture elements of the complexity of interactions, including those that are difficult to verbalise (Cristancho & Helmich, 2019), and (b) advanced multilevel methods that include different characteristics of contexts and individuals.

Another limitation concerns the challenges of analysing moderated mediation models with categorical independent variables (i.e., supervision styles). The interpretation of regression coefficients, based on difference scores between supervision styles, is less straightforward. This would have required visualisation methods if moderation or moderated mediation effects were found. Furthermore, the relatively small sample size may have led to small effects being missed. Our moderated mediation models produced 15 (model 8) and 12 (model 15) estimated coefficients due to the dummy codes of our supervision styles. Although unlikely, it remains possible that individual differences in work-related need strength might slightly modify the effects of supervision styles on need-based experiences and affective states of junior doctors.

### Future research

Future research may disentangle the underlying reasons for differences in perception and appraisal of supervision styles on psychological need satisfaction and frustration in clinical practice. Although work-related need strength did not explain these differences, other individual (trait or state) differences between junior doctors and characteristics of learning contexts and cultures may provide additional insights. Need-based experiences are dynamic, can differ between situations (social interactions), domains (contexts), and general level (e.g., individual differences in causality orientations), and can change over time (Ryan & Deci, 2017). Longitudinal research on supervisory interactions between junior doctors and consultants can examine if and how behavioural patterns develop. Based on these patterns junior doctors may adopt coping mechanisms and, for example, resilience (Neufeld & Malin, 2022; Waterschoot et al., 2020) to deal with positive and negative experiences that influence their basic psychological needs. Drawing on SDT, it can also be hypothesised that junior doctors’ causality orientations may explain additional variance in need-based experiences. People with an autonomy orientation tend to interpret contextual cues as more autonomy supportive, while people with a control orientation tend to interpret these cues as more controlling (Ryan & Deci, 2017). Furthermore, characteristics of the learning culture of medical specialties and the relationship between junior doctors and supervisors (Watling et al., 2014) may explain additional variance in need-based experiences and their outcomes. The possible role of (implicit) values towards learning and development was not tested in this research, but this may have influenced the values that junior doctors integrate during PGME. Finally, more practical examples of need supporting and thwarting experiences may help to refine how and why junior doctors’ need-based experiences occur. This is important for developing interventions that reduce need frustration and promote need fulfilment in clinical practice.

Secondly, future research is needed to examine (de)motivating supervision styles in actual clinical settings. A recent paper by Neufeld (2025) provides some practical examples of how clinicians can support the individual psychological needs of students. It seems relevant to explore to which extent need support (or frustration) of all three needs tends to co-occur in practice. For instance, when supervising consultants provide volition and choice to junior doctors, do they also tend connect with and display confidence in junior doctors’ capabilities to perform well? Contrastingly, if supervising consultants control junior doctors’ autonomous behaviour, do they also tend to act cold towards them and display distrust in junior doctors’ ability to perform well? Additionally, it is of practical relevance to understand how and why supervising consultant apply different (de)motivating styles. This may shed further light on the relevance of contextual aspects that may play a role that may prevent or stimulate motivating supervision focused on junior doctors’ needs.

## Conclusions

In this experimental vignette study, individual differences in work-related need strength did not modify the effects of (de)motivating supervisions styles on need satisfaction, need frustration, and affective states. These findings support SDT’s principle of universality that need satisfaction leads to positive outcomes, while need frustration leads to negative outcomes. However, generalisability of these findings to the dynamics and complexities of actual clinical practice needs to be addressed in future research. Nevertheless, our findings show the importance of providing need-supportive supervision and avoid need-thwarting supervision to foster junior doctors’ affective states. Future research may refine how, why, and when these need-based experiences occur during supervisory interactions.

## Electronic supplementary material

Below is the link to the electronic supplementary material.

Supplementary Material 1

## Data Availability

Due to regulations and restrictions related to the confidentiality of participants, data are not publicly available. Requests for data can be addressed to the programme leader of LEARN (learn@umcg.nl). Requests will be evaluated on a case-by-case basis.
