# Supplementary material for: Does need strength influence the impact of supervision styles on junior doctors’ affective states?
Source: Adv Health Sci Educ Theory Pract. 2025 Dec 1;31(4):1089–111. doi: 10.1007/s10459-025-10491-y (PMC13553623; doi:10.1007/s10459-025-10491-y)
Supplement: Supplementary file 1 [file 10459_2025_10491_MOESM1_ESM.pdf]

Does need strength influence the impact of supervision styles on junior doctors' affective states?

Advances in Health Sciences Education

Wieke E. van der Goot[1,2], Robbert J. Duvivier[1,3]

[1]University of Groningen, University Medical Center Groningen, Wenckebach Institute for Education and Training (WIOO), Lifelong Learning, Education and Assessment Research Network (LEARN), Groningen, the Netherlands

[2]Martini Hospital, Martini Academy, Groningen, the Netherlands

[3]Parnassia Psychiatric Institute, The Hague, the Netherlands

Corresponding author: w.e.van.der.goot@rug.nl

Supplementary Information 1

Manipulation Checks

To test our experimental manipulations, we performed two subsequent 2 (need support: *high* versus *low*) x 2 (directiveness: *high* versus *low*) analyses of variance (ANOVA). Obviously, the findings are practically identical to van der Goot et al. (2025). However, negligible differences in the outcomes of the manipulation checks occurred due to small differences in the outlier analyses. These analyses with affective states resulted in the exclusion of one respondent that was included in van der Goot et al. (2025). In addition, another respondent that was excluded in van der Goot et al. (2025) was included in the present study. Thus, the total number of participants in both studies is the same.

The first 2 x 2 ANOVA on the need-support manipulation check revealed a strong main effect of need support, $F(1, 146) = 472.28$, $p < .001$, $\eta_p^2 = .76$. Participants in the *high* need-support conditions (M = 5.61, SD = 0.95) perceived more need support than participants in the *low* need-support conditions (M = 2.36, SD = 0.89), indicating a successful manipulation of need support[1].

The second 2 x 2 ANOVA on the directiveness manipulation check revealed a strong main effect of directiveness, $F(1, 146) = 144.44$, $p < .001$, $\eta_p^2 = .50$. Participants in the *high* directiveness conditions (M = 5.15, SD = 0.99) perceived more directiveness than participants in the *low* directiveness conditions (M = 3.01, SD = 1.38). Hence, we concluded that the experimental manipulation of directiveness was successful, too.[2]

---

[1] The main effect of directiveness was not significant, $F(1, 146) = 0.59$, $p = .44$, $\eta_p^2 = .004$, but the interaction effect between need support and directiveness was significant, $F(1, 146) = 4.82$, $p = .03$, $\eta_p^2 = .03$. Follow-up ANOVA with post hoc (Bonferroni) tests indicated that the difference in perceived need support was larger in the *low* directiveness conditions ($M_{\text{High NS, Low DIR}} = 5.74$, $SD_{\text{High NS, Low DIR}} = 0.69$ versus $M_{\text{Low NS, Low DIR}} = 2.15$, $SD_{\text{Low NS, Low DIR}} = 0.79$, $p < .001$) than the *high* directiveness conditions ($M_{\text{High NS, High DIR}} = 5.53$, $SD_{\text{High NS, High DIR}} = 1.10$ versus $M_{\text{Low NS, High DIR}} = 2.60$, $SD_{\text{Low NS, High DIR}} = 0.95$, $p < .001$). However, there were no significant differences in need support between the two *high* need-support conditions ($p = 1.00$) nor between the two *low* need-support conditions ($p = .24$).

[2] Again, we found a significant interaction effect between need support and directiveness, $F(1, 146) = 14.15$, $p = .001$, $\eta_p^2 = .09$. Follow-up ANOVA with post hoc (Bonferroni) tests indicated that the difference in perceived directiveness was larger in the *low* need support conditions ($M_{\text{Low NS, High DIR}} = 4.87$, $SD_{\text{Low NS, High DIR}} = 1.03$ versus $M_{\text{Low NS, Low DIR}} = 2.22$, $SD_{\text{Low NS, Low DIR}} = 1.16$, $p < .001$) than the *high* need support conditions ($M_{\text{High NS, Low DIR}} = 3.97$, $SD_{\text{High NS, Low DIR}} = 0.96$ versus $M_{\text{High NS, High DIR}} = 5.36$, $SD_{\text{High NS, High DIR}} = 0.92$, $p < .001$). The directiveness scores of the two *low* directiveness conditions differed significantly, $p = .001$, but the scores of the two *high* directiveness conditions did not, $p = .233$. Note that the main effect of need support was significant as well, $F(1, 146) = 44.05$, $p < .001$, $\eta_p^2 = .23$. That is, participants in the *high* need support conditions (M = 4.78, SD = 1.16) perceived more directiveness than participants in the *low* need support conditions (M = 3.46, SD = 1.72).

Supplementary Information 2

The PROCESS macro (Hayes, 2022) is a statistical tool, based on Ordinary Least Squares (OLS) regression (for continuous outcome variables) or maximum likelihood logistic regression (for dichotomous dependent variables), that estimates coefficients which best describe the linear relationship between independent (predictors) and dependent (outcome) variables. The tool allows testing of simple to highly complex mediation, moderation and moderated mediation models in which multiple variables are added to best describe the relationship between the independent (X), mediating (M), moderating (W), and dependent (Y) variables. The output of the tool includes estimated parameters that partition the effect of X on Y through one or more mediating and/or moderating variables. Furthermore, the tool also allows for bootstrapping procedures and correcting violations of the homoscedasticity of error variances assumption. We used a heteroscedasticity-consistent estimator (HC4; Cribari-Neto,2004) because some of the mediator variables had heteroscedasticity of the variances. Using HC4 results in the same coefficients, but creates less biased and more robust error variances and confidence intervals. We compared the error variances and confidence intervals between the HC4 corrected estimates and the bootstrapped estimates. The outcomes were quite similar and did not affect the significant or non-significant findings of our tested models. Thus, we show only the bootstrapped estimates in the Tables and combined this with *p*-values that were calculated based on the coefficients with HC4-correction. The next paragraphs illustrate the graphical and statistical representation of the models that were used in this study to test our hypotheses.

In *Hypothesis 1* we tested whether the effects of supervision styles (X) on positive and negative affect (Y1 and Y2) were mediated through psychological need satisfaction (M1) and need frustration (M2) by using Model 4 of Hayes' PROCESS Macro (Hayes, 2022). This model predicts the unique effects of all added mediating variables by partitioning the effect of X on Y through the indirect effects (*ab* coefficients) and the remaining direct effect (c'). See Figure SI2_1 and Figure SI2_2.

Fig. SI2_1 Graphical representation of Model 4 (based on Hayes, 2022) – with two parallel mediators

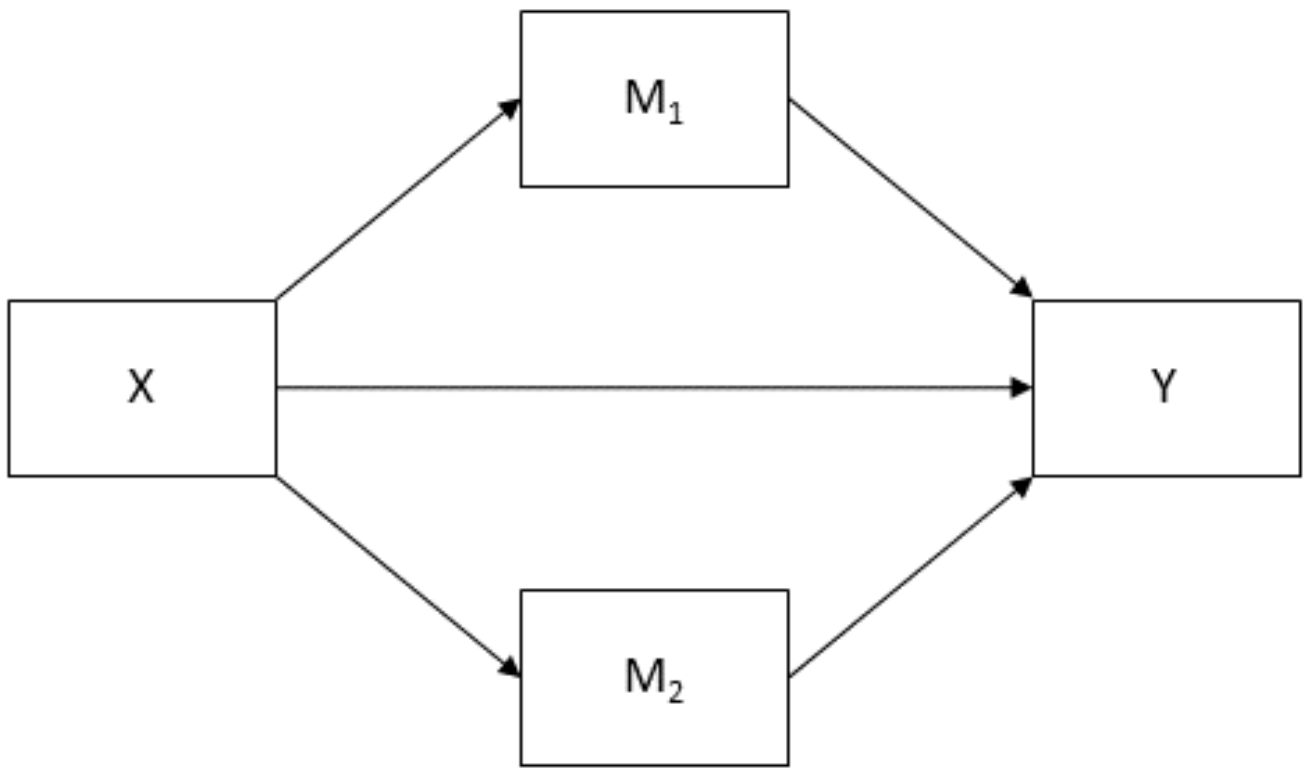

Fig. SI2_2 Statistical representation of Model 4 with a categorical independent variable and two parallel mediators

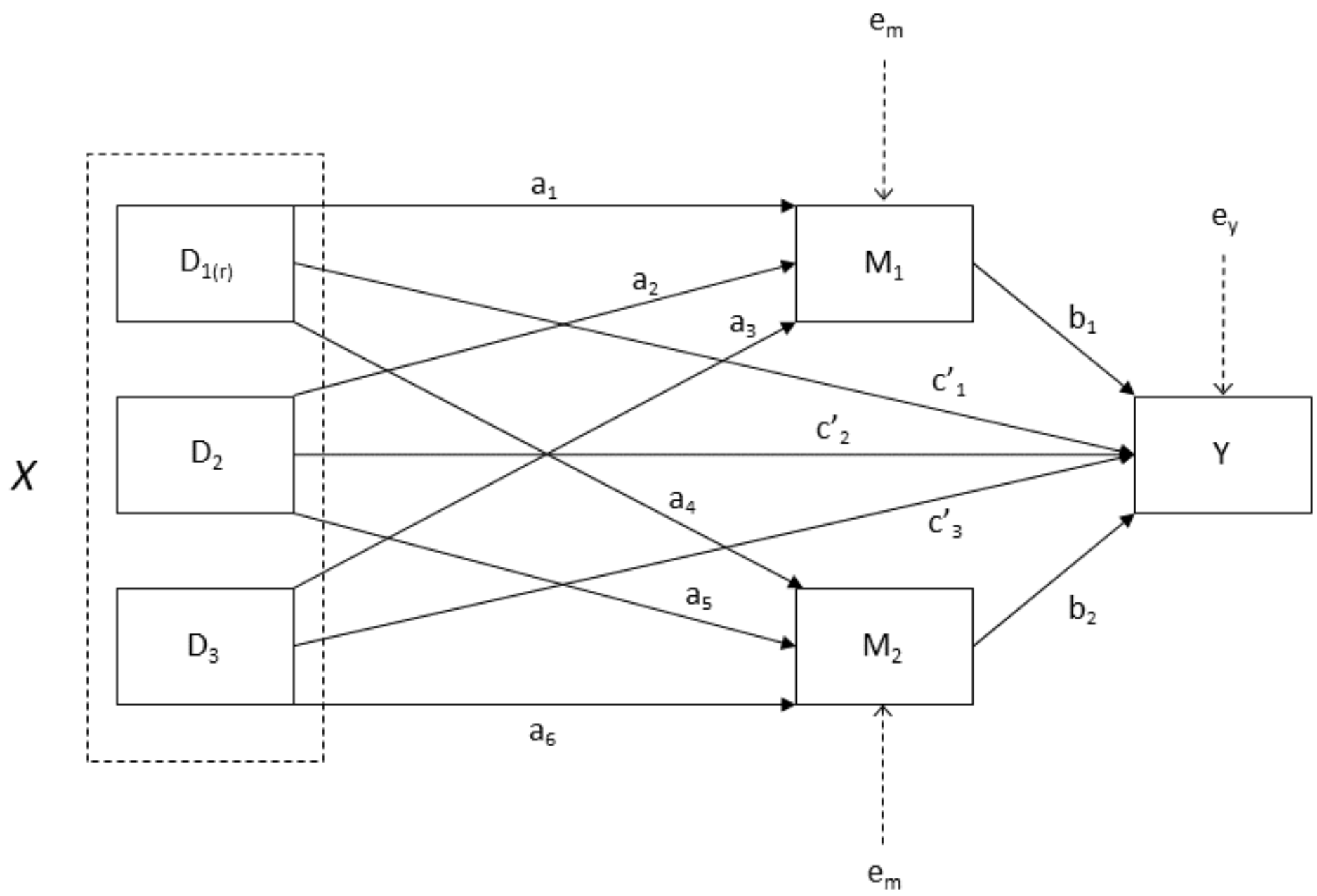

Note. X = independent variable 'supervision styles' recoded into 3 dummy variables (D_1-D_3), D_{1r} = reversed dummy code. M1 & M2 = mediator variables, Y = outcome variable 'affective state'; $a_{1\text{-}6}$ = estimated direct effects of X on M; $b_{1\text{-}2}$ = estimated direct effects of M on Y; $c'_{1\text{-}3}$ = estimated direct effects of X on Y, independent of the effect of M on Y.

In *Hypothesis 2* we tested whether need strength moderated the effect of the corresponding need domain on affective state. For this we used a conditional process model, Model 15, to test direct and/or indirect effect of an independent variable X on a dependent variable Y, with one moderator (W) and one

mediator (M). This model of second stage moderation tests if the mediating effect of M on Y and X on Y is moderated by W (see Figure SI2_3 and Figure SI2_4).

Fig. SI2_3 Graphical representation of Model 15 (based on Hayes, 2022) – 2nd stage moderated mediation

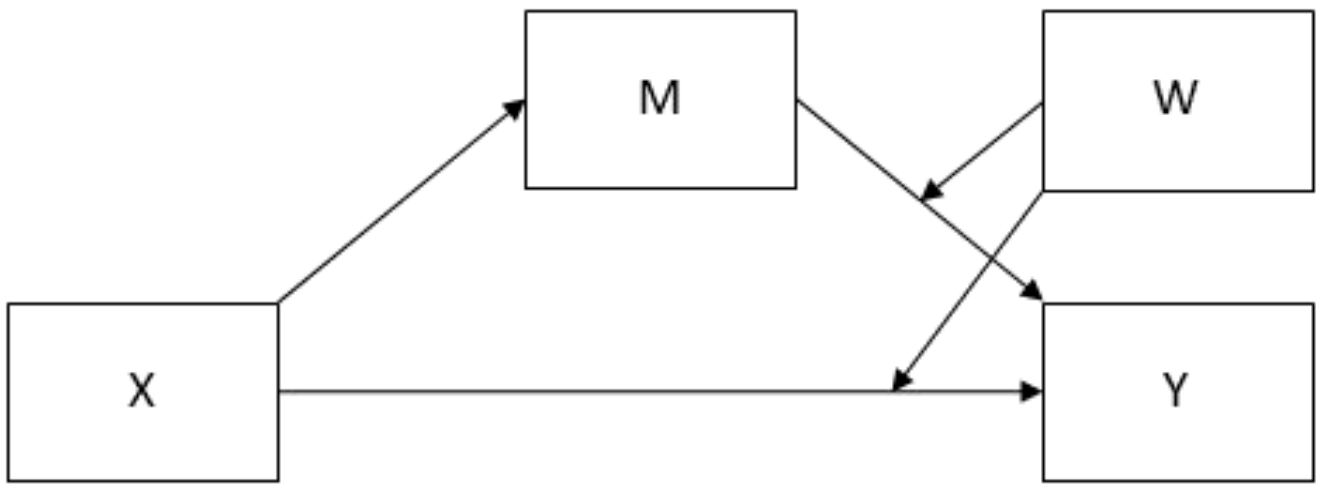

Fig. SI2_4 Statistical representation of Model 15 with a categorical independent variable

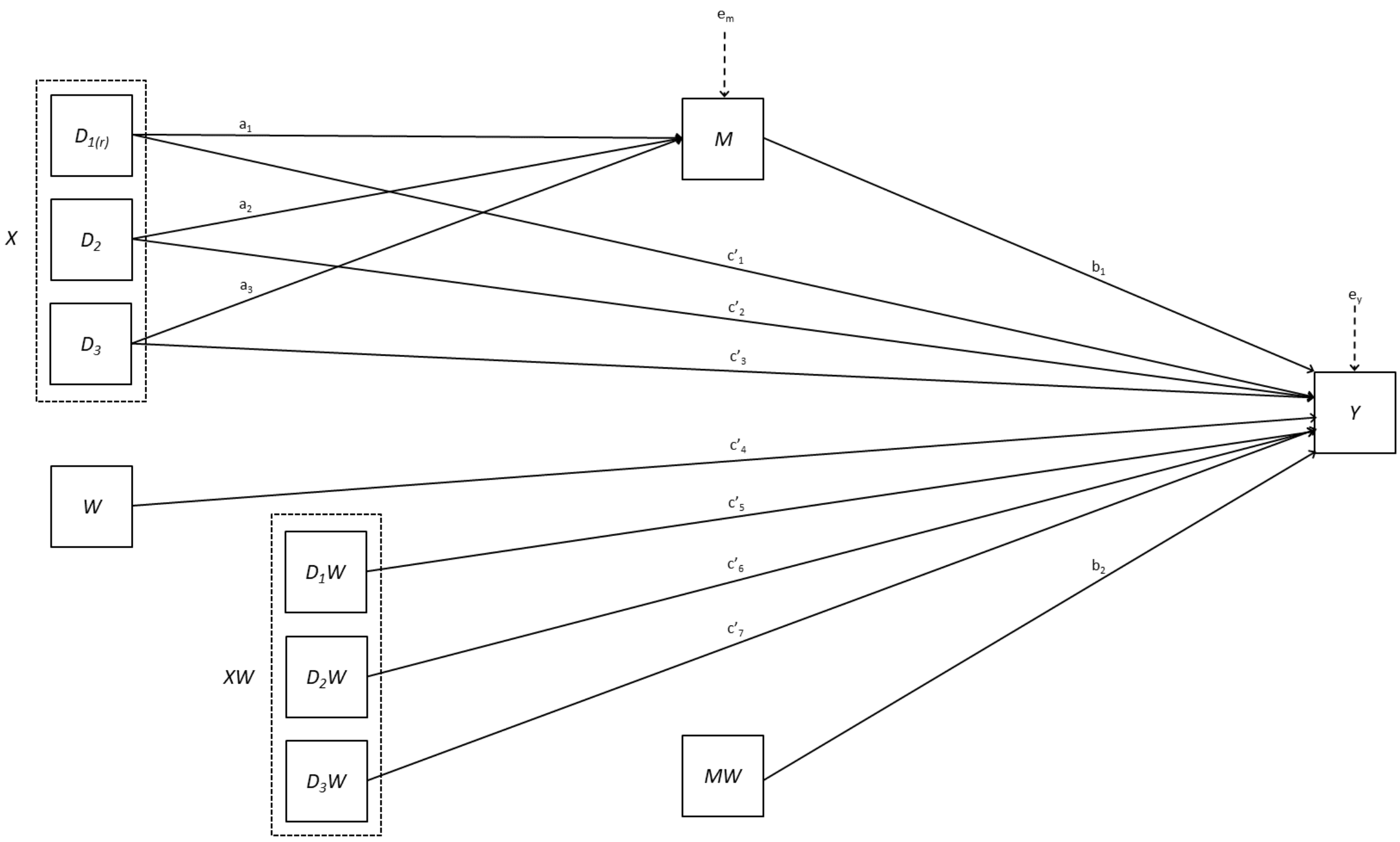

Note. X = independent variable 'supervision styles' recoded into 3 dummy variables (D_1-D_3), D_{1r} = reversed dummy code. M = mediator variables, Y = outcome variable 'affective state'; $a_{1\text{-}3}$ = estimated direct effects of X on M; b_1 = estimated direct effect of M on Y; b_2 = estimated direct effect of the product of MW on Y; $c'_{1\text{-}7}$ = estimated direct effects of X, W, and the product of XW on Y, independent of the effect of M on Y.

In *Hypothesis 3* we tested whether need strength moderated the effect of the corresponding need domain on affective state. For this we used a conditional process model, Model 8, to test direct and/or

indirect effect of an independent variable X on a dependent variable Y with one moderator (W) and one mediator (M). This model of first stage moderation tests if the effect of X on M and Y is moderated by W (see Figure SI2_5 and Figure SI2_6).

Fig. SI2_5 Graphical representation of Model 8 (based on Hayes, 2022) – 1st stage moderated mediation

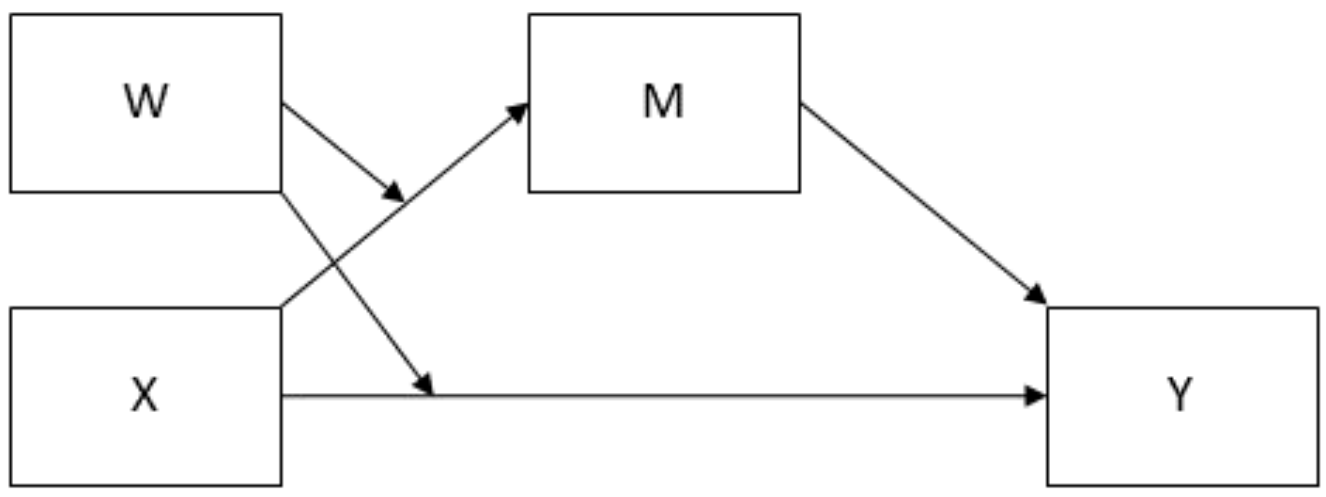

Fig. SI2_6 Statistical representation of Model 8 with a categorical independent variable

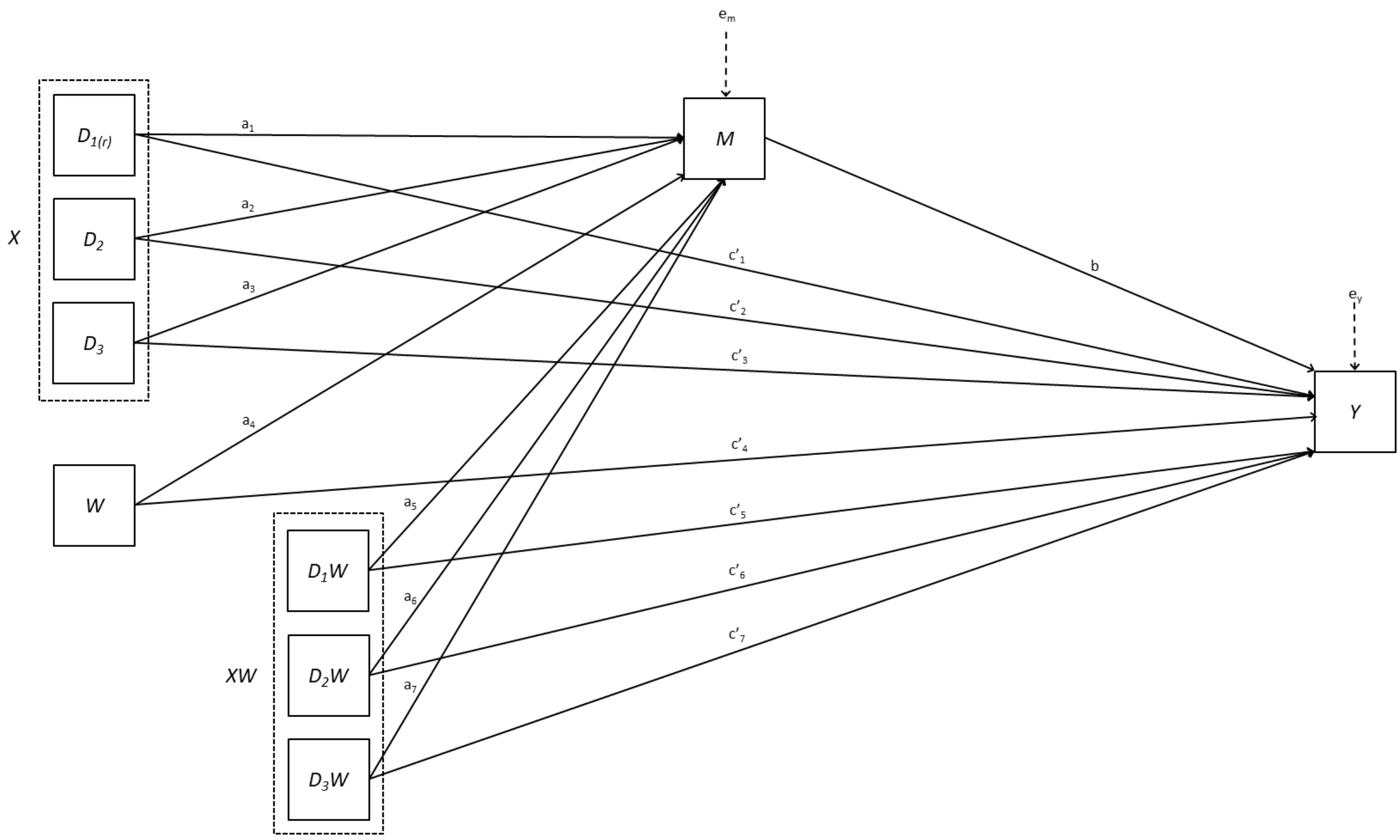

Note. X = independent variable 'supervision styles' recoded into 3 dummy variables (D_1-D_3), D_{1r} = reversed dummy code. M = mediator variables, Y = outcome variable 'affective state'; $a_{1\text{-}7}$ = estimated direct effects of X , W, and the product of XW on M; b = estimated direct effect of M on Y; $c'_{1\text{-}7}$ = estimated direct effects of X, W, and the product of XW on Y, independent of the effect of M on Y.

Supplementary Information 3

Table SI3

Indirect and Direct Effects of Supervision Styles on Affective States through Need Satisfaction and Need Frustration (Simple Parallel Mediation Models).

		Relative indirect effects										Direct effect				R^2
		Need Satisfaction					Need Frustration					Affective state				
Antecedent		Coeff.	*SE*	95% CI			Coeff.	*SE*	95% CI			Coeff.	*SE*	95% CI		
				LL	*UL*				*LL*	*UL*				*LL*	*UL*	
Positive affect																.51***
D_1	a_1b_1	0.40**	0.15	0.12	0.69	a_4b_2	0.16	0.13	-0.10	0.39	c'_1	0.41*	0.16	0.09	0.73	
D_2	a_2b_1	0.08	0.05	0.00	0.20	a_5b_2	-0.02	0.02	-0.07	0.01	c'_2	0.07	0.11	-0.16	0.28	
D_3	a_3b_1	0.05	0.05	-0.05	0.15	a_6b_2	0.03	0.03	-0.03	0.10	c'_3	0.31*	0.13	0.06	0.58	
Negative affect																.79***
$D_{1\ reversed}$	a_1b_1	0.10	0.10	-0.09	0.31	a_4b_2	0.88***	0.12	0.65	1.12	c'_1	0.60***	0.14	0.33	0.89	
D_2	a_2b_1	-0.02	0.02	-0.08	0.02	a_5b_2	0.11	0.08	-0.04	0.25	c'_2	0.08	0.10	-0.11	0.28	
D_3	a_3b_1	-0.01	0.02	-0.07	0.02	a_6b_2	-0.15	0.11	-0.38	0.06	c'_3	-0.03	0.12	-0.28	0.21	

Note. All regression coefficients are unstandardised. A heteroscedasticity consistent standard error estimator (HC4) was used in the analyses and to calculate the *p*-values. The standard error (SE) and 95% confidence intervals (CI) are bootstrapped with 10,000 samples. *LL* = lower limit; *UL* = upper limit. D_1 = difference between *high* and *low* need supportive supervision styles; D_2 = difference between both *high* need supportive supervision styles; D_3 = difference between both *low* need supportive styles; $D_{1\ reversed}$ = difference between *low* and *high* need supportive supervision styles. R^2 represents the explained variance of the tested model. Due to multiple testing, we used a Bonferroni correction and considered $p < .02$ as significant. *$p < .02$ **$p < .01$ *** $p < .001$

Supplementary Information 4

Table SI4

2 x 2 MANOVA Results with Need Support (High Versus Low) and Directiveness (High Versus Low) as Between-Subject Factors

	Multivariate *F*(8,139)	V			Univariate *F*(1,146)	η_p^2
Need support	39.71***	.70	1.	Autonomy satisfaction	67.39***	.32
			2.	Competence satisfaction	185.63***	.56
			3.	Relatedness satisfaction	94.49***	.39
			4.	Autonomy frustration	126.42***	.46
			5.	Competence frustration	116.28***	.44
			6.	Relatedness frustration	110.28***	.43
			7.	Positive affect	102.70***	.41
			8.	Negative affect	225.26***	.61
Directiveness	2.80**	.14	1.	Autonomy satisfaction	6.38*	.04
			2.	Competence satisfaction	3.48	.02
			3.	Relatedness satisfaction	0.30	.00
			4.	Autonomy frustration	2.23	.02
			5.	Competence frustration	3.25	.02
			6.	Relatedness frustration	0.07	.00
			7.	Positive affect	7.29**	.05
			8.	Negative affect	0.02	.00
Need support x	2.28*	.12	1.	Autonomy satisfaction	0.42	.00
Directiveness			2.	Competence satisfaction	0.17	.00
			3.	Relatedness satisfaction	0.86	.00
			4.	Autonomy frustration	0.09	.00
			5.	Competence frustration	8.82**	.06
			6.	Relatedness frustration	3.41	.02
			7.	Positive affect	1.92	.01
			8.	Negative affect	3.08	.02

Note. *$p < .05$. **$p < .01$. ***$p < .001$. V = Pillai's Trace (because Box's M was significant).

Table SI4 shows the anticipated multivariate main effect of need support. In addition, we also found a small multivariate main effect of directiveness, $F(8, 139) = 2.80$, $p = .007$, $\eta_p^2 = .14$. This effect could be attributed at the univariate level to autonomy satisfaction and positive affect. Participants in the *low* directiveness conditions scored higher on autonomy satisfaction than participants in the *high* directiveness conditions ($M_{\text{DIR LOW}} = 3.29$, $SD_{\text{DIR LOW}} = 0.87$ versus $M_{\text{DIR HIGH}} = 3.09$, $SD_{\text{DIR HIGH}} = 0.96$, $p = .013$). Positive affect showed a similar pattern ($M_{\text{DIR LOW}} = 3.33$, $SD_{\text{DIR LOW}} = 0.67$ versus $M_{\text{DIR HIGH}} = 3.19$, $SD_{\text{DIR HIGH}} = 0.83$, $p = .008$). The small multivariate interaction effect, $F(8, 139) = 2.28$, $p = .025$, $\eta_p^2 = .12$, could be attributed to competence frustration. Participants in the *low* need support, *high* directiveness condition ($M_{\text{NS LOW, DIR HIGH}} = 3.27$, $SD_{\text{NS LOW, DIR HIGH}} = 1.00$) scored significantly higher on competence frustration than all other conditions ($M_{\text{NS HIGH, DIR LOW}} = 1.52$, $SD_{\text{NS HIGH, DIR LOW}} = 0.69$ versus $M_{\text{NS HIGH, DIR HIGH}} = 1.36$, $SD_{\text{NS HIGH, DIR HIGH}} = 0.63$; $M_{\text{NS LOW, DIR LOW}} = 2.61$, $SD_{\text{NS LOW, DIR LOW}} = 1.01$), *ps* < .001.
